# An expression signature of 19 human endogenous retroviruses identifies immunogenic luminal breast cancers likely to respond to immunotherapy

**DOI:** 10.3389/fonc.2026.1728115

**Published:** 2026-06-16

**Authors:** Silvia Mihaela Ilie, Julie Lecuelle, François Ghiringhelli, Caroline Truntzer, Sylvain Ladoire

**Affiliations:** 1 Department of Medical Oncology, Georges Francois Leclerc Cancer Center, Dijon, France; 2 Cancer Biology Transfer Platform, Georges Francois Leclerc Cancer Center, Dijon, France; 3 INSERM UMR 1231, Center for Translational and Molecular Medicine (CTM), Dijon, France; 4 University Burgundy Europe, Dijon, France

**Keywords:** breast cancer, endogenous retroviruses, immunology, immunotherapy, viral mimicry

## Abstract

**Background:**

Human endogenous retroviruses (HERVs) are single-stranded RNA viruses that have integrated into the human germline, and whose sequences represent approximately 8% of the human genome. In cancer cells, certain HERV sequences can be re-expressed, thereby promoting an innate and adaptive immune response, particularly through the induction of an interferon response in tumors, known as “viral mimicry”. We sought to elucidate the links between HERV expression and tumor immunogenicity in different biological subtypes of early breast cancer (eBC).

**Patients and methods:**

We used publicly available RNA-seq gene expression data of breast cancer samples and corresponding matched normal data from The Cancer Genome Atlas (TCGA). HERV sequences were detected and quantified using Telescope software. For each patient, we computed an overall HERV expression and a signature of HERVs whose expression is associated with biological signals of intra-tumor viral mimicry. Correlations between these expression signatures, signs of tumor immunogenicity, and patient prognosis were examined.

**Results:**

Overall HERV tumor load varies little among the different entities of eBC and does not appear to impact tumor immunogenicity, regardless of the biological subtype. In contrast, restricting the analyses to HERVs whose expression is associated with viral mimicry hallmarks, it was possible to identify a signature of 19 HERVs whose expression is associated with signs of tumor immunogenicity. Moreover, this “Breast HERV19” expression signature was associated with better prognosis in HR^+^HER2^−^ eBC, in which its expression was also correlated with biomarkers of sensitivity to anti PD-1 immunotherapy, such as tumor-infiltrating lymphocytes (TILs), PD-L1, and estrogen receptor (ER) expression levels.

**Conclusion:**

In eBC, our “Breast HERV19” expression signature makes it possible to isolate a subgroup of HR^+^HER2^−^ tumors presenting strong immunogenicity, better prognosis, and biological characteristics that suggest a possible benefit from immunotherapy.

## Introduction

Human endogenous retroviruses (HERVs) are single-stranded RNA (ssRNA) viruses that have integrated into the human germline by means of their long terminal repeats (LTRs). They constitute genomic elements that are the remnants of these ancestral germline infections and retrotranspositions (1).

HERV-related sequences represent approximately 8% of the human genome (2), are distributed in approximately 700,000 elements, and are generally epigenetically repressed in normal human tissues. Indeed, most HERVs are highly degenerated through evolutionary pressure, including accumulation of mutations or homologous recombination between proviral LTRs (3). However, some HERVs can be re-expressed in tumor cells (3–5) (compared with corresponding healthy tissue), although the involvement of this phenomenon in oncogenesis has not been formally demonstrated (6). In addition to this potential role in carcinogenesis, this abnormal expression may also promote both innate and adaptive immune response against tumor cells, either by creating a pool of new tumor-associated antigens (7–10) or by inducing an interferon response in tumor cells (termed “viral mimicry”) resembling a response against viral double-stranded RNA (dsRNA), consistent with that of virus-infected cells (4, 11). Re-expression of certain HERV sequences has been described in many types of human cancer, including breast cancer (12, 13), but the possible link between this cellular phenomenon and the immunogenicity of breast cancer is poorly described.

Immunotherapy [mainly targeting PD-(L)1] is currently undergoing considerable development in oncology, particularly for the treatment of certain breast cancers, such as triple-negative (TN) subtypes, where, in combination with chemotherapy, it improves overall patient survival in both advanced (14) and early (15) stages. However, the development of immunotherapy in other breast cancer subtypes [luminal tumors expressing hormone receptors (HRs) and tumors with HER2 amplification] is much less advanced, because the benefit of this therapeutic approach in the overall patient population is lower. There is therefore a major medical need to identify patients whose tumors are most likely to respond to immunotherapy. At present, with the exception of metastatic TN, where the level of PD-L1 expression on immunohistochemistry (IHC; CPS score) is used to select patients who are candidates for the combination of pembrolizumab (an anti-PD-1 agent) with first-line chemotherapy, no biomarker is used in routine clinical practice in breast cancer to guide immunotherapy treatment.

Our aim in this study was to evaluate, in the different biological subtypes of early breast cancer (eBC), both the overall expression of HERVs in tumors (overall HERV tumor load) and that of HERVs associated with different pathways related to viral mimicry/interferon response, in order to assess their association with (i) biological hallmarks of tumor immunogenicity and (ii) the long-term prognosis of patients.

Our results show that in eBC, in contrast to the global HERV tumor load, the study of the expression of a shared signature of 19 HERVs associated with viral mimicry (“Breast HERV19”) makes it possible to isolate a subgroup of luminal tumors presenting strong immunogenicity, a better prognosis, and biological characteristics that suggest a possible benefit from immunotherapy.

## Results

### The overall expression of HERVs selectively expressed in early breast cancers (overall HERV tumor load) differs little according to the different biological subtypes of disease, except in HR^+^HER2^−^ tumors

We analyzed publicly available RNA-seq gene expression data from *N* = 971 tissue samples (*N* = 36 from normal healthy tissue of breast and *N* = 935 from eBC of different subtypes), coming from The Cancer Genome Atlas (TCGA) database (16). The complete clinical–biological characteristics of the patients and tumors analyzed are shown in **Table 1**. Briefly, of the *N* = 935 tumors analyzed, the majority (*N* = 608 (65%) expressed estrogen HRs on IHC, without HER2 oncogene amplification (HR^+^HER2^−^ subtype), while *N* = 157 (16.8%) had HER2 amplification (HER2 amplified subtype), and *N* = 170 (18.2%) did not express HRs and had no HER2 amplification (TN subtype). In addition to overall analyses of all tumors, or according to their IHC-based subtype, we also analyzed TNs according to their transcriptomic subtype (17) [basal-like immune-activated (BLIA), basal-like immune-suppressed (BLIS), luminal androgen receptor (LAR), and mesenchymal (MES)]. Similarly, for HR^+^HER2^−^ tumors, we also carried out analyses according to their transcriptomic subtype given by the ER^+^ tumor intrinsic subtype classification (18) [luminal A (Lum A), luminal B (Lum B), HER2 enriched, or basal-like]. The numbers of these different biological entities are also given in **Table 1**.

**Table 1:** Clinical characteristics of the study population (whole cohort, HR+HER2-, HER2 amplified and TN cohorts).

Variable	All N = 935	HR+HER2- N = 608	HER2 amplified N = 157	TN N = 170	P-value	Adjusted p-value
Sex					–	–
Female	935 (100%)	608 (100%)	157 (100%)	170 (100%)		
Age	58 (49, 67)	59 (49, 68)	58 (48, 67)	54 (47, 63)	**0.002**	**0.004**
≤60	280 (30%)	168 (28%)	48 (31%)	64 (38%)	**0.041**	**0.047**
>60	655 (70%)	440 (72%)	109 (69%)	106 (62%)		
IHC subtype					–	–
HER2 amplified	157 (16.8%)	–	–	–		
HR^+^HER2^-^	608 (65%)	–	–	–		
TN	170 (18.2%)	–	–	–		
ER^+^ tumors intrinsic subtypes*					**-**	**-**
Basal Like	17 (2.8%)	17 (2.8%)	–	–		
HER2 enriched	5 (0.8%)	5 (0.8%)	–	–		
Lum A	427 (70%)	427 (70%)	–	–		
Lum B	159 (26%)	159 (26%)	–	–		
NA	327	0	157	170		
Triple negative intrinsic subtypes**					–	–
BLIA	45 (28%)	–	–	45 (28%)		
BLIS	51 (31%)	–	–	51 (31%)		
LAR	34 (21%)	–	–	34 (21%)		
MES	32 (20%)	–	–	32 (20%)		
Unknown	773	608	157	8		
ER status					**<0.001**	**<0.001**
Negative	216 (23%)	0 (0%)	46 (29%)	170 (100%)		
Positive	719 (77%)	608 (100%)	111 (71%)	0 (0%)		
PR status					**<0.001**	**<0.001**
Negative	323 (35%)	94 (15%)	68 (43%)	161 (95%)		
Positive	612 (65%)	514 (85%)	89 (57%)	9 (5.3%)		
HER2 status					**<0.001**	**<0.001**
Non amplified	778 (83%)	608 (100%)	0 (0%)	170 (100%)		
Amplified	157 (17%)	0 (0%)	157 (100%)	0 (0%)		
T stage					**0.030**	**0.04**
T1	241 (26%)	173 (29%)	30 (19%)	38 (22%)		
T2-T4	691 (74%)	433 (71%)	127 (81%)	131 (78%)		
Unknown	3	2	0	1		
N stage					**<0.001**	**<0.001**
N0	442 (48%)	274 (46%)	59 (39%)	109 (64%)		
N1	309 (34%)	208 (35%)	60 (39%)	41 (24%)		
N2	108 (12%)	71 (12%)	23 (15%)	14 (8.2%)		
N3	58 (6.3%)	41 (6.9%)	11 (7.2%)	6 (3.5%)		
Unknown	18	14	4	0		

Comparisons were performed between HR^+^HER2^-^, HR^-^HER2^+^ and TN tumors.

HR; Hormone Receptor, HER2; Human Epidermal growth factor 2, TN; Triple Negative, IHC; Immunohistochemical, Lum A; Luminal A, Lum B; Luminal B, BLIA; Basal-Like Immune-Activated, BLIS; Basal-Like Immune-Suppressed, LAR; Luminal Androgen Receptor, MES; Mesenchymal, ER; Estrogen Receptor, PR; Progesterone Receptor.

*This classification was only performed on HR^+^HER2^-^ tumors.

**This classification was only performed on TN tumors.

Bold values indicate statistically significant results (p < 0.05).

**Figure 1:**
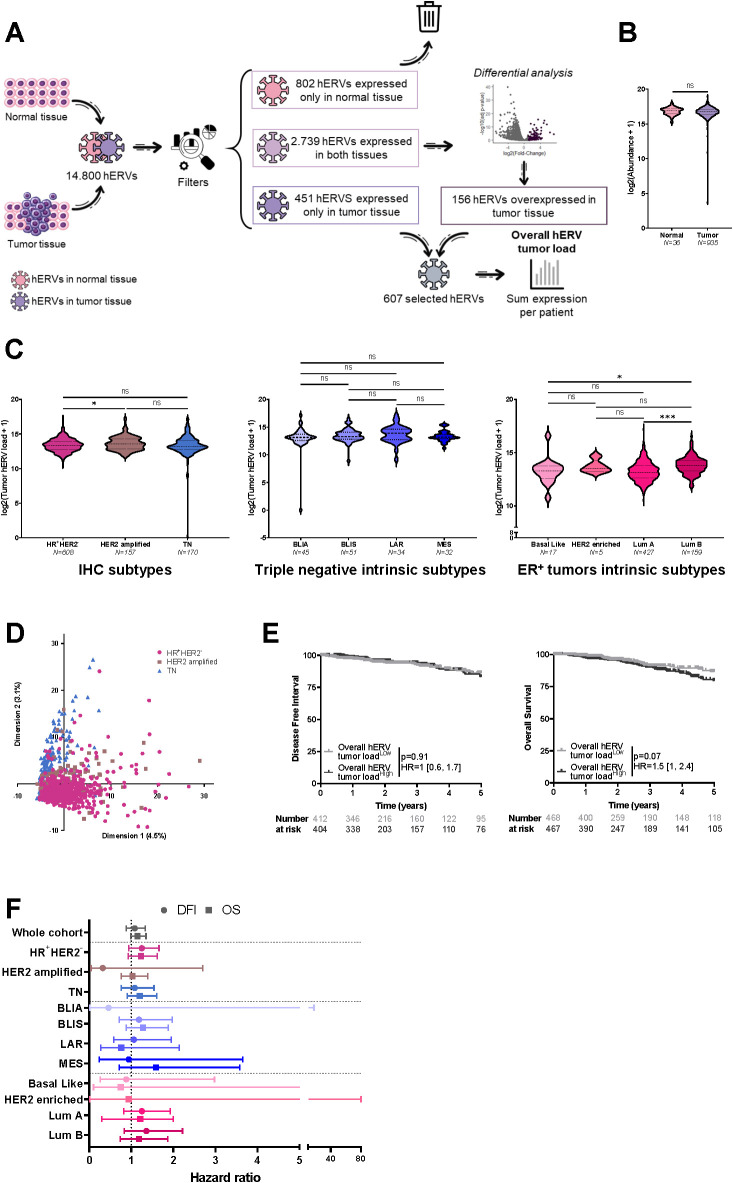
**(A)** Flowchart of HERV selection. **(B)** Violin plots showing abundance of HERVs selected in normal and tumor tissues. **(C)** Violin plots showing the overall HERV tumor load expression according to IHC subtype (left), triple-negative intrinsic subtypes (middle), and ER^+^ tumor intrinsic subtypes (right). **(D)** Principal component analysis plot of patients using the 607 selected HERVs. Patients are colored according to IHC subtypes. **(E)** Kaplan–Meier curves with patients stratified according to tumor HERV load status (High vs. Low, using median as threshold) for disease-free interval in the left panel, and overall survival in the right panel. **(F)** Forest plots representing hazard ratios and confidence intervals for Cox models for disease-free interval (round points) and overall survival (square points) estimated using overall HERV tumor load expression in the whole cohort, and according to IHC subtypes, triple-negative, and ER^+^ tumor intrinsic subtypes. For BLIA patients, only DFI was shown as there were not enough events for OS. The same applies to HER2 enriched patients with DFI. Ns, non-significant, *p < 0.05, **p < 0.01 and ***p < 0.001. IHC, immunohistochemistry; ER, estrogen receptor; TN, triple negative; BLIA, basal-like immune-activated; BLIS, basal-like immune-suppressed; LAR, luminal androgen receptor; MES, mesenchymal; Lum A, luminal A; Lum B, luminal B.

Our first objective was to analyze the role of overall HERV expression in eBC: for each patient/sample, the sum of expression of all HERVs [keeping only those whose expression was greater than five reads for at least 5% of cases (**Figure 1A**)] was analyzed first and showed no difference between tumor samples, compared with normal breast tissue samples (**Figure 1B**).

For this reason, we then considered for overall expression analysis only those HERVs that were more selectively expressed in breast tumors, namely, those expressed only in tumor samples (*N* = 451) or HERVs whose expression was significantly higher in tumors than in normal breast tissue (*N* = 156). Finally, we focused our analyses of the consequences of overall HERV expression (overall HERV tumor load) on these *N* = 607 tumor-selected HERVs (**Figure 1A**).

We showed that overall expression of tumor HERVs differed very little between the different breast cancer subtypes given by IHC (**Figures 1C**, left), with only HR^+^HER2^−^ tumors having a tumor HERV load that was slightly significantly lower than HER2-amplified tumors [median = 13.4 and interquartile range (IQR) = 1.2 for HR^+^HER2^−^ and 13.5 (1.4) for HER2-amplified; *p* = 0.04]. Among TN tumors, there was also no difference in overall HERV tumor load between the different transcriptomic tumor subtypes (**Figure 1C**, middle). In HR^+^HER2^−^ tumors, Lum B intrinsic subtype tumors had a significantly higher overall HERV tumor load [13.8 (0.98)], compared to basal-like subtype tumors [13.3 (0.93), *p* = 0.03], and Lum A tumors [13.2 (4.2), *p* < 0.001] (**Figure 1C**, right).

Taken together, these results therefore seem to indicate that in eBC, the overall expression of HERVs selectively expressed in tumor tissue varies relatively little between the different biological tumor entities defined by IHC. For HR^+^HER2^−^ tumors, the intrinsic Lum B subtypes appear to have the highest overall HERV tumor load.

### In early breast cancer samples, overall HERV tumor load has no prognostic impact on survival and does not appear to have any impact on tumor immunogenicity, whatever the biological tumor subtype

As previously shown, the overall expression of tumor HERVs does not show significant differences between the different breast cancer subtypes defined by IHC. Similarly, in general, the expression profile of these *N* = 607 selected tumor HERVs did not distinguish different tumor clusters, as shown by unsupervised principal component analysis (**Figure 1D**).

We then assessed whether overall HERV tumor load could be associated with the prognosis of eBC. Taking into account the median expression of overall HERV tumor load, survival analyses showed that neither disease-free interval (DFI, **Figure 1E** left) nor overall survival (OS, **Figure 1E** right) appeared to be influenced by overall HERV tumor load in the whole population. The results were the same within the different biological entities of breast cancer, although there seemed to be a tendency for HR^+^HER2^−^ tumors to have poorer survival in patients with a tumor with high overall HERV tumor burden (**Figure 1F**), with no clear difference within the different transcriptomic subtypes of luminal tumors (particularly between Lum A and Lum B tumors).

Despite the absence of a clear prognostic impact of overall HERV tumor load, we nevertheless investigated whether this biological signature could be associated with hallmarks of tumor immunogenicity and/or signs of intra-tumor immune response. To examine this hypothesis, we studied the association between the expression of overall HERV tumor load and the expression level of *N* = 16 expression signatures either linked to the presence of immune cell populations [particularly myeloid and lymphoid cells: signatures myeloid dendritic cells, monocytic lineage, B-cell lineage, NK cells, cytotoxic lymphocytes, CD8 T cells, T cells, and tumor-infiltrating lymphocytes (TILs) (see the Patients and methods section)] or related to the presence of a cytotoxic T-cell response and/or the expression of immune checkpoint inhibitors [signatures CYTOX, Th1, CTL, ICK, and CD274 (see the Patients and methods section)].

**Figure 2:**
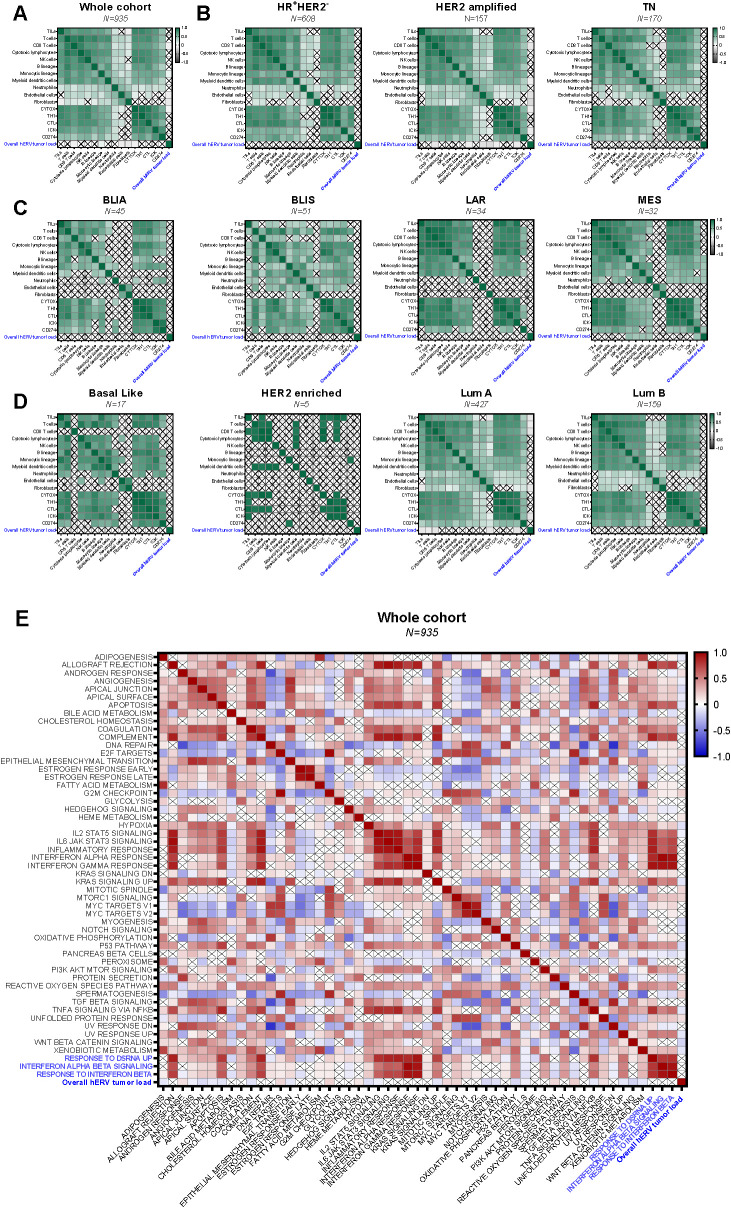
**(A–D)** Heatmaps of Pearson’s correlation values between overall HERV tumor load, abundance of MCP-counter cell populations, and immune signatures in **(A)** the whole cohort and according to **(B)** IHC subtypes, **(C)** triple-negative intrinsic subtypes, and **(D)** ER^+^ tumor intrinsic subtypes. Non-significant *p*-values are represented by a white crossed cell. **(E)** Heatmap of Pearson’s correlation values between overall HERV tumor load, HALLMARK signaling pathways, and three interferon pathway expressions in the whole cohort. IHC, immunohistochemistry; ER, estrogen receptor; TN, triple negative; BLIA, basal-like immune-activated; BLIS, basal-like immune-suppressed; LAR, luminal androgen receptor; MES, mesenchymal; Lum A, luminal A; Lum B, luminal B.

In the whole cohort of eBC cases, we found that signatures assessing the presence of myeloid/lymphoid cell populations, as well as those related to the presence of a cytotoxic T-cell response and/or the expression of immune checkpoint inhibitors, significantly correlated with each other (**Figure 2A**). In contrast, the level of expression of overall HERV tumor load did not correlate significantly with any of these signatures of tumor immunogenicity and/or signs of intra-tumor immune response (**Figure 2A**). Similar results were obtained in each breast cancer subtype defined by IHC (HR^+^HER2^−^, HER2 amplified and TN subtypes: **Figure 2B**). Among the different transcriptomic subtypes of TN (BLIA, BLIS, LAR, and MES), the pattern of correlation was the same, again with no correlation between overall HERV tumor load expression and the different immune signatures (**Figure 2C**). The same was true for the different transcriptomic subtypes of HR^+^HER2^−^ tumors (basal-like, HER2-enriched, Lum A, and Lum B) (**Figure 2D**).

Taken together, these results indicate that the overall HERV tumor load is probably not a relevant biomarker in breast cancer for exploring the potential immunogenic role (and indirectly the possible prognostic role) of human endogenous retroviruses.

### Unlike overall HERV tumor load, which is not correlated with cellular pathways associated with interferon response, restricting the analyses to HERVs whose expression is associated with hallmarks of viral mimicry makes it possible to identify a signature of 19 HERVs whose expression is associated with signs of tumor immunogenicity

Given the potential link between re-expression of certain HERVs in tumor cells and the ability of the latter to initiate in turn a potential interferon response, we examined whether the overall HERV tumor load was ultimately associated with certain cell signaling pathways (particularly concerning viral mimicry, such as the interferon alpha and beta cell response, or the response to double-stranded DNA). We thus confirmed that, in fact, overall HERV tumor load is not globally correlated with any classical cell signaling pathway (**Figure 2E**) (last line on the heatmap). In particular, there was no correlation between overall HERV tumor load and expression of cell signaling signatures related to the viral mimicry cell response (response to interferon alpha/beta, or response to double-stranded DNA, marked in blue in **Figure 2E**). Similar results were observed irrespective of the IHC-based subtype of breast cancer, and irrespective of the transcriptomic subtype of TN, or HR^+^HER2^−^ tumor, when cell signaling pathway expression was represented with patients ordered according to the overall HERV tumor load (**Supplementary Figure 1**).

These results therefore seem to show that not all HERVs selectively expressed or significantly more highly expressed in breast tumors have the capacity to generate significant viral mimicry. We therefore set out to identify the HERVs (among the *N* = 607 HERVs included in the overall HERV tumor load) whose expression was significantly correlated with the three Molecular Signature Database (MSigDB) cell signaling pathways associated with viral mimicry (GEISS_RESPONSE_TO_DSRNA_UP, REACTOME_INTERFERON_ALPHA_BETA_SIGNALING, and GOBP_RESPONSE_TO_INTERFERON_BETA).

**Figure 3:**
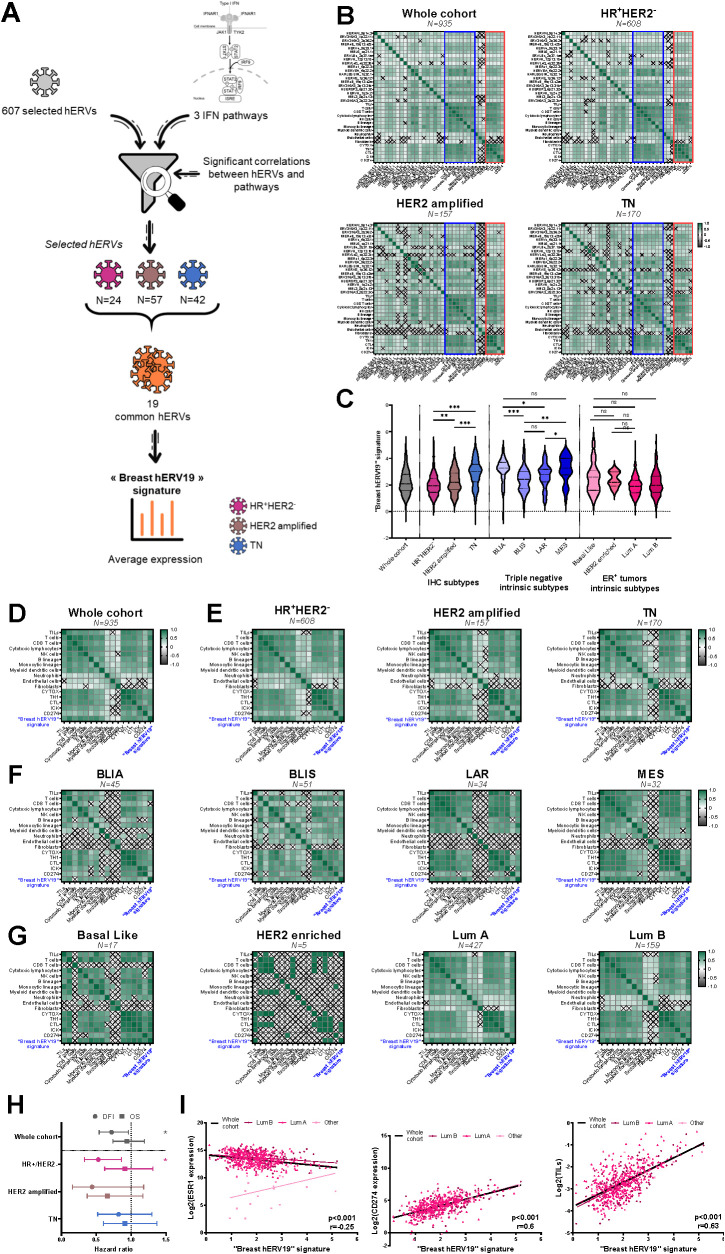
**(A)** Flowchart of the “Breast HERV19” signature’s creation. **(B)** Heatmaps of Pearson’s correlation coefficient values between expression of the HERVs selected for the “Breast HERV19” signature, abundance of MCP-counter cell populations, and immune signatures in the whole cohort and according to IHC subtypes; non-significant *p*-values are represented by a white crossed cell. **(C)** Violin plots showing expression of the “Breast HERV19” signature in the whole cohort and according to IHC subtypes, triple-negative, and ER^+^ tumor intrinsic subtypes. (**D–G**) Heatmaps of Pearson’s correlation coefficient values between “Breast HERV19” signature expression, abundance of MCP-counter cell populations, and immune signatures in **(D)** the whole cohort, and according to **(E)** IHC subtypes, **(F)** triple-negative intrinsic subtypes, and **(G)** ER^+^ tumor intrinsic subtypes. Non-significant *p*-values are represented by a white crossed cell. **(H)** Forest plots representing hazard ratios and confidence intervals for univariate Cox models for disease-free interval (round points) and overall survival (square points) estimated using “Breast HERV19” signature expression in the whole cohort and IHC subtypes. **(I)** Scatter plot of “Breast HERV19” signature expression and ESR1, CD274, and TILs gene expression (left, middle, and right panels, respectively) in HR^+^HER2^−^ patients. Points are colored according to ER^+^ tumor intrinsic subtype (other = Basal Like or HER2 enriched). Linear regression was computed for each subtype and for the whole cohort (black line). Pearson’s correlation coefficient values in the whole cohort are indicated for each panel. Ns, non-significant, *p < 0.05, **p < 0.01, and ***p < 0.001. IHC, immunohistochemistry; ER, estrogen receptor; TN, triple negative.

We retained only those HERVs whose expression was significantly positively correlated with the expression of these three cells’ signaling signatures: among the 607 initial HERVs, we thus identified *N* = 77 HERVs in the overall cohort of tumor samples, including *N* = 57 in HR^+^HER2^−^ tumors, *N* = 24 in HER2-amplified tumors, and *N* = 42 in TN, meeting these criteria. In order to generate a signature of HERVs that could be applied to all the different subtypes of breast cancer, we retained only those HERVs whose expression was significantly positively correlated with the three viral mimicry signaling pathways and in the three subtypes of breast cancer, which made it possible to isolate *N* = 19 HERVs in this situation (**Figure 3A**; **Supplementary Figure 2**) (HERVW_5p14.3, ERV316A3_Xp22.11, ERV316A3_2q36.2, MER4B_19q13.42b, HERV4_5q33.1, HML6_4q21.1, ERVLB4_2q37.1a, HERVH_12p13.1b, HERVL40_4q32.3b, MER41_6p22.3, HERVEA_5q22.2, HARLEQUIN_1q32.1, HERVE_1p36.12, MER4B_19q13.42a, ERV316A3_3q13.31b, HUERSP3_6p21.32, HERVH_1q24.2, HML3_8q24.13, and ERV316A3_2q22.3c). For further analysis, we used the average expression of these 19 HERVs as the “Breast HERV19” signature.

Each of these 19 HERVs showed variable levels of positive correlation with expression signatures either related to the presence of myeloid and lymphoid cell populations (**Figure 3B**, blue rectangles) or related to the presence of a cytotoxic T response and/or the expression of immune checkpoint inhibitors (**Figure 3B**, red rectangles) previously explored, irrespective of IHC-based breast cancer subtype. When these 19 HERVs were grouped together to form a global expression signature, it could be seen that the expression of the “Breast HERV19” signature varied greatly according to the biological subtype of breast cancer: the “Breast HERV19” signature was significantly more expressed in TNs [median = 3 (IQR = 1.3)] than in HER2-amplified [2.2 (1.2)] and HR^+^HER2^−^ [1.9 (1)] tumors (*p* < 0.001 for both; **Figure 3C**). Within TNs, there were also differences between the transcriptomic subtypes, with a higher level of expression of the “Breast HERV19” signature in the BLIA and MES subtypes. In contrast, within HR^+^HER2^−^ tumors, there was no difference between the different transcriptomic subtypes, particularly between Lum A and Lum B tumors (**Figure 3C**).

By selecting these HERVs on the basis of their ability to associate with viral mimicry signals, their grouping into a global expression within the “Breast HERV19” signature showed that the level of expression of this signature was very strongly correlated with the expression signatures linked to the presence of myeloid and lymphoid cell populations, or linked to the presence of a cytotoxic T response and/or the expression of immune checkpoint inhibitors (**Figures 3D–G**). This strong correlation can be observed for each of the immune signatures evaluated, both in the overall population (**Figure 3D**) and in each of the biological entities of breast cancer, whether defined by IHC (**Figure 3E**), by transcriptomics for TN (**Figure 3F**), or by HR^+^HER2^−^ tumors (except for the HER2-enriched subtype, probably because of the very low number of cases, *N* = 5) (**Figure 3G**).

Collectively, these results therefore demonstrate the biological relevance of restricting the analysis to HERVs whose expression is associated with hallmarks of viral mimicry, in order to identify those whose expression is associated with signs of tumor immunogenicity.

### The “Breast HERV19” expression signature is associated with better prognosis in early breast cancer, particularly for HR^+^HER2^−^ subtypes in which the expression of this signature is also correlated with biomarkers of sensitivity to anti-PD-1 immunotherapy

In recent years, the biological hallmarks of immune response in tumors have been associated with better prognosis in breast cancer, particularly in TN [specifically with regard to the presence of TILs (19)]. We therefore set out to examine whether our “Breast HERV19” expression signature could also be prognostic in eBC. By examining both the DFI and OS of patients in our cohort, we showed that the greater the expression of the “Breast HERV19” signature, the better the DFI (**Figure 3H**). This relationship was found in the overall cohort of patients, but was essentially linked to HR^+^HER2^−^ tumors. Each of the *N* = 19 HERVs included in our expression signature had an association of different weight with DFI: for exploratory purposes, the statistical relationship between the expression of each of the 19 HERVs and DFI is given for the overall cohort and HR^+^HER2^−^ tumors in **Supplementary Table 1**. Importantly, for TN and HER2-amplified subtypes, despite a trend in the same direction, there was no statistically significant association between expression of the “Breast HERV19” signature and DFI. Regarding OS, whether for the overall cohort or for each of the different subtypes, there was no association with the expression of the “Breast HERV19” signature (**Figure 3H**).

As the prognosis of eBC is multifactorial, we then examined the independent prognostic value of our HERV expression signature in the overall cohort, and more specifically in HR^+^HER2^−^ cancers. Univariate and multivariate Cox models showed an independent prognostic role (on DFI) of the expression of the “Breast HERV19” signature, both in the overall cohort of patients and especially in the population of patients with HR^+^HER2^−^ eBC (**Table 2**). By multivariate analysis, for this group of patients, expression of the “Breast HERV19” signature remained associated with longer DFI (HR = 0.50 [0.30–0.80]; *p* = 0.006), independently of the other clinico-biological variables classically associated with prognosis in HR^+^HER2^−^ eBC (age at diagnosis, T stage, N stage, Ki67 expression, and transcriptomic subtype) (**Table 2**).

**Table 2:** Cox models for disease-free interval in the whole cohort and in patients with HR+HER2- tumors.

Variables	Whole cohort *n=806*		HR^+^HER2^-^ *n=525*	
	Univariate	Multivariate	Univariate	Multivariate
“Breast hERV19” signature	0.7 [0.5, 1]; **0.047**	0.6 [0.5, 0.9]; **0.01**	0.5 [0.3, 0.9]; **0.009**	0.5 [0.3, 0.8]; **0.006**
KI67 expression	1 [1, 1]; 0.2	1 [1, 1]; 0.11	1 [0.99, 1]; 0.47	1 [1, 1]; 0.8
Age				
<50	**-**	–	–	–
≥50	0.6 [0.4, 1.1]; 0.08	0.7 [0.4, 1.1]; 0.13	0.88 [0.43, 1.81]; 0.72	1 [0.5, 2.2]; 0.9
T stage				
T1	**-**	–	–	–
T2-T4	2.7 [1.3, 5.6]; **0.01**	2 [0.9, 4.3]; p=0.09	5.2 [1.6, 17.2]; **0.006**	4 [1.2, 13.6]; **0.03**
N stage				
N0	**-**	–	–	–
N1	1.6 [0.8, 3]; 0.16	1.4 [0.7, 2.6]; 0.35	2.1 [0.9, 4.9]; 0.08	1.7 [0.7, 3.9]; 0.2
N2	3.1 [1.5, 6.6]; **0.003**	2.4 [1.1, 5.2]; **0.02**	3.8 [1.4, 10.2]; **0.008**	2.9 [1, 8.2]; **0.04**
N3	4.8 [1.9, 12]; **<0.001**	4.3 [1.6, 11.1]; **0.003**	4.2 [1.1, 15.8]; **0.03**	3.5 [0.9, 13.8]; 0.07
ER+ tumors intrinsic subtypes*				
Lum A	**-**	–	–	–
Lum B	**-**	–	1.5 [0.7, 3.2]; 0.28	1.4 [0.6, 3.3]; 0.5
Other	**-**	–	1.7 [0.4, 7.4]; 0.46	1.7 [0.3, 9.1]; 0.5

Results are described as Hazard Ratio (HR), with 95% confidence interval (CI) and p-value (p); (HR [CI]; p).

*This classification was only performed on HR^+^HER2^-^ tumors; so it was not included in the Cox analysis of the whole cohort.

Bold values indicate statistically significant results (p < 0.05).

Our “Breast HERV19” expression signature therefore appears to have a strong prognostic role in HR^+^HER2^−^ eBC, a subtype in which it is also strongly associated with signs of anti-tumor immune response. Immunotherapy (in particular anti-PD-1) has developed significantly in recent years for the treatment of early and metastatic TN (14, 15). It is possible that these immunotherapy treatments could also improve the management of certain HR^+^HER2^−^ breast cancers, since recent clinical results have indicated that pembrolizumab and nivolumab (two anti-PD-1 agents), in combination with chemotherapy, could improve the proportion of patients with pathological complete response (pCR) following preoperative treatment (20, 21). Although there is no validated biomarker for selecting patients with HR^+^HER2^−^ tumors who are likely to benefit from immunotherapy, it nevertheless seems that in these pivotal clinical trials, tumors heavily infiltrated with TILs, expressing PD-L1, or weakly expressing estrogen receptors (ERs) benefit most from neoadjuvant immunotherapy by anti-PD-1 (20, 21). We therefore investigated whether the expression of our “Breast HERV19” signature could be associated with these emerging biomarkers for the selection of HR^+^HER2^−^ tumors for treatment by immunotherapy. Our results showed that expression of the “Breast HERV19” signature was significantly positively correlated (*p* < 0.001) with expression of the *CD274* gene (coding for the PD-L1 protein) and with expression of the RNA signature TILs score (22) (*p* < 0.001), and negatively correlated with expression of the *ESR1* gene (coding for the ER), whatever the intrinsic subtype of HR^+^HER2^−^ eBC (**Figure 3I**).

These results show not only that our “Breast HERV19” expression signature is strongly associated with signs of tumor immunogenicity, but also that it is independently associated with better prognosis in HR^+^HER2^−^ eBC, a subtype in which it could also contribute, along with other biomarkers, to better identify tumors that respond to anti PD-1 immunotherapy strategies.

## Discussion

In this study, we examined the potential immunogenic role of the expression of certain HERVs in the different biological subtypes of breast cancer. Our study shows that it is probably not relevant to study the overall expression of all HERVs in tumors (overall HERV tumor load), since this does not seem to be associated with signs of tumor immunogenicity, whatever the biological subtype of eBC considered. For this reason, in a second phase, we selected only HERVs whose tumor expression was associated with signs of viral mimicry. This signature of expression of 19 HERVs, common to all the different types of breast cancer, not only is strongly associated with numerous hallmarks of tumor immunogenicity (immune cell infiltration, cytotoxic T response, and expression of inhibitory immune checkpoints), but also constitutes an independent prognostic factor in HR^+^HER2^−^ tumors, in which the expression of this signature seems to be associated with several biomarkers reported as being predictive of the benefit of anti-PD-1 immunotherapy.

The vast majority of HERVs have degenerated proviral genomes, which prevents their transcription in normal tissues. However, some may have retained their open reading frames (ORFs) intact in the host genome, allowing them to be transcribed, and therefore enabling the expression of certain viral proteins within the cellular proteome (3). Although expression of HERVs remains low in healthy tissues (5), it can increase to variable levels in cancer cells as a result of the epigenetic deregulation that accompanies carcinogenesis. One of the best-described examples is the high expression of ERVE-4 in clear cell renal cell carcinomas (ccRCCs), tumors in which there is significant epigenetic deregulation, leading in particular to hypomethylation of the 5′LTR region of ERVE-4, which has a response element to hypoxia-inducible transcription factor 2α (HIF-2α), a transcription factor present in excess in ccRCC cells due to inactivation of the tumor suppressor gene VHL (Von Hippel Lindau) (23). Such a mechanistic link from a biological point of view has not yet been demonstrated in breast cancer.

To the best of our knowledge, no study has specifically explored the overall expression of HERVs in the different subtypes of breast cancer (defined by IHC or transcriptomics), and the association between this biological parameter and tumor immunogenicity. Previous studies on human tumors have shown that overall HERV tumor load can be associated with biological signs of viral mimicry (24), particularly in colon and pancreatic cancers. The divergent results of our study, conducted specifically in breast cancer, therefore suggest that this parameter is likely to have a different influence on tumor immunogenicity depending on the type of cancer studied.

In breast cancer, the majority of studies have focused solely on certain HERVs or certain groups of HERVs, such as the HERVK family, which have been reported several times to be overexpressed in breast cancer compared with normal breast tissue (13, 25–27), with signs of humoral or cellular immune response against these HERVs in some studies (26, 28, 29). On the other hand, our study is the only one to our knowledge to have evaluated the overall HERV tumor load specifically in breast cancers and, thus, to show that, even considering only the expression of HERVs selectively expressed in tumors, this global parameter is not associated with biological signs of tumor immunogenicity, or with a different prognosis for patients, regardless of the biological subtype of breast cancer. The results of the first part of our work therefore seem to show that the epigenetic processes enabling greater, or selective, expression of certain HERVs in breast cancers result in a global phenomenon involving numerous HERVs whose expression has no immunological consequences in the tumors. More generally, our results seem to show that beyond the association with signs of immune response, overall HERV tumor load does not appear to influence intracellular signaling pathways in tumors. These results undoubtedly explain the absence of consequences of overall HERV load on the prognosis of the patients whose tumors we analyzed. It therefore seems that overall HERV tumor load is not a relevant biological parameter for breast cancer.

The biological links between tumor expression of HERVs and immunogenicity in human cancers are still largely unknown. The biological hypothesis supporting this link is that non-self proteins of viral origin can potentially be recognized by the host immune system and stimulate the adaptive immune response: T and B responses against certain retroviral antigens have been demonstrated in humans and animals (26, 30, 31), as have cytotoxic CD8^+^ T responses against HERVs antigens in certain cancer patients, some of whom have shown a tumor response (7–10, 26).

Furthermore, ssRNA, dsRNA, and dsDNA, produced by abnormally active HERVs in tumor cells, can act as pathogen-associated molecular patterns (PAMPs) and can be recognized by cytoplasmic nucleic acid sensors, such as retinoic acid-inducible gene I (RIG-I), melanoma differentiation-associated protein 5 (MDA-5), Toll-like receptors (TLRs) and cyclic GMP-AMP synthase (cGAS), which serve as specific pattern recognition receptors (PRRs) (32, 33), to trigger innate immune responses through mimicking those of exogenous viruses (33). This “viral mimicry” is accompanied by the induction of type I and III interferon signaling, thereby increasing cellular immunogenicity (34, 35). For this reason, we felt that it was biologically relevant, for the second part of our work, to restrict our analyses to HERVs whose expression was significantly associated with the activity of cellular signaling pathways involved in viral mimicry. By doing this, we were able to considerably reduce the number of HERVs (to *N* = 19) that appear to have an impact on the immunogenicity of breast cancers. We show that HERVs associated with viral mimicry signatures differ between breast cancer biological subtypes, suggesting slightly different epigenetic deregulation between different breast cancer biological entities. However, we show that some of the HERVs that appear to be immunogenic are common to all breast cancer subtypes, and on this basis, we combined them into a common expression signature (pan-breast cancer) of *N* = 19 HERVs. Expression of this “Breast HERV19” signature differed according to the biological subtype of breast cancer, with, interestingly, a higher level of expression in the TN subtype, which is the subtype in which immunotherapy has so far shown the most interest. It should be noted that among TNs, the BLIA subtype, which is the most immunogenic (17), was also the one where expression of the “Breast HERV19” signature was highest.

The biological singularity of this 19-HERV subset, compared to the bulk of HERVs expressed in breast tumors, likely lies in their preserved capacity to generate immunostimulatory nucleic acid intermediates (notably dsRNA species) able to engage cytoplasmic PRRs (RIG-I, MDA-5, cGAS-STING, and TLRs). Indeed, these 19 HERVs were specifically selected on the basis of a significant positive correlation of their expression with three independent transcriptional readouts of viral mimicry/IFN signaling (response to dsRNA, IFN-α/β signaling, and response to IFN-β), and across all three IHC-defined breast cancer subtypes. This convergent association strongly suggests that, contrary to the majority of HERVs whose re-expression appears to have no immunological consequence, the elements composing the “Breast HERV19” signature retain sequence and structural features compatible with the production of immunogenic transcripts (e.g., partial ORFs, intact LTRs allowing bidirectional transcription, or complementary sequences favoring dsRNA folding), thereby triggering the innate antiviral cellular machinery. Functional *in vitro* studies will be needed to formally demonstrate this mechanistic uniqueness.

However, it is in luminal tumors (HR^+^HER2^−^) that our results seem most interesting (despite a lower overall expression of the signature compared with the other subtypes): firstly, to our knowledge, this is the first study to show an independent prognostic role for the expression of certain HERVs in breast cancer in such a large cohort of patients. Moreover, luminal tumors have historically been considered to be of little or no immunogenicity, with very few studies of immunotherapy for the treatment of this type of tumor. However, very recently, two randomized clinical trials showed that, in patients with HR^+^HER2^−^ eBC, the addition of anti PD-1 immunotherapy to neoadjuvant chemotherapy resulted in a significantly higher rate of pCR (20, 21). However, this benefit of immunotherapy in luminal tumors is not observed in all patients, and it therefore seems very important to be able to select patients for this treatment. In these two large randomized trials (CheckMate 7FL with nivolumab and KEYNOTE 756 with pembrolizumab as anti-PD-1), subgroup analyses showed that patients who benefited most from the addition of immunotherapy were mainly those with tumors expressing low levels of ER, tumors richly infiltrated with TILs, and tumors strongly expressing PD-L1 (20, 21). Our analyses show that not only is the expression of the “Breast HERV19” signature strongly positively correlated with signs of tumor immunogenicity (high infiltration of myeloid/lymphoid cells, presence of a cytotoxic T response, and/or expression of immune checkpoint inhibitors), but it is also statistically associated with the three biomarkers, making it possible to identify the population of patients with a luminal tumor in whom anti-PD-1 immunotherapy appears to be of most interest. The possibility that the expression of certain HERVs may favor responses to immunotherapy has been suggested by clinical observations showing that increased expression of HERVs could be associated with responses to immunotherapy in patients treated for urothelial cancer (36) or kidney cancer (29), but, to date, not for breast cancer, to the best of our knowledge. Our observations on hormone-dependent luminal eBC are all the more interesting since estradiol and progesterone have been reported to increase HERV-K (HML-4) env (37) and HERV-K (HML-4) RT transcripts as well as HERV-K (HML-4) RT protein levels (38) in breast cancer cell lines. Collectively, these results, including our own, provide insights into the links between modulation of the estrogen pathway and expression of HERVs. Endocrine therapies used in luminal breast cancers could therefore also have an influence on the expression profile of tumor HERVs.

The observations made in our work are also interesting because epigenetic modulator treatments such as DNA methyltransferase inhibitors (DNMTis) can be used in the treatment of cancers with the aim of therapeutically modulating tumor epigenetics, particularly to reactivate inappropriately silenced tumor suppressor genes. Such treatments could also potentially promote viral mimicry, via the re-expression of immunogenic HERVs. Several molecules in this class have been shown to be capable of inducing the production of HERV dsRNA, thereby stimulating the cellular biological cascade at the origin of an interferon response (4, 11). Other pharmacological strategies such as inhibition of histone deacetylation complexes, lysine demethylases, and histone methyltransferases (39, 40) have also been shown to induce viral mimicry in various types of tumor (4, 11, 41).

Thus, promising preclinical results suggest that HERV-induced viral mimicry brought about through inhibition of DNMT1 or the histone demethylase can synergize with immune checkpoint inhibitors (4, 11, 41). Interestingly, preclinical data suggest that CDK4/6 inhibitors (which are widely used in association with endocrine therapy in patients with advanced or early HR^+^HER2^−^ breast cancer) are capable of blocking DNMT1 expression, which induces viral mimicry in relation to the increased expression of certain HERVs in tumor cells and a therapeutic synergy between abemaciclib and anti-PD-L1 immunotherapy (42).

From a practical standpoint, we agree that HERV quantification by RNA-seq is not yet broadly available in routine clinical practice. In our cohort, expression of the “Breast HERV19” signature was strongly and positively correlated with both the transcriptomic TIL score and CD274 (PD-L1) expression, particularly in HR^+^/HER2^–^ tumors. This suggests that, in the short term, immune infiltration assessment (TILs on H&E) and PD-L1 IHC could serve as accessible surrogate biomarkers, capturing a substantial portion of the immunogenic signal that our HERV signature reflects. However, our results also indicate that the HERV signature remains independently associated with DFI in multivariate analyses, implying that it captures additional biological information beyond what TILs or PD-L1 alone provide. Therefore, while TILs/PD-L1 represent a feasible first step for clinical translation, the development of simpler, targeted assays (e.g., RT-qPCR panels or NanoString-based readouts) restricted to the 19 HERVs of our signature would represent a more direct and potentially more informative biomarker strategy.

Our work suffers from a number of limitations: firstly, we were unable to obtain an independent cohort of HR^+^HER2^−^ tumor samples for external validation of our results obtained in the TCGA cohort. In addition, our conclusions regarding the predictive role of the “Breast HERV19” signature on the benefit of immunotherapy are purely hypothetical, since we have no analyses of tumors from patients who have received this treatment.

However, our original results provide new data on the potential immunogenic role of HERVs in breast cancer, particularly in HR^+^HER2^−^ tumors. Our expression signature for 19 HERVs makes it possible to isolate a subgroup of luminal tumors with high immunogenicity, better prognosis, and biological and clinical characteristics that suggest a benefit from immunotherapy, and it warrants validation in independent datasets to confirm its clinical relevance.

## Patients and methods

### Study population

Nine hundred and thirty-five patients with eBC for which RNA-seq was available from TCGA (https://www.cancer.gov/tcga, accessed date: v24.0, 7 May 2020 for clinical data and v32.0, 29 March 2022 for RNA-seq in GDC data portal) were considered in this study. Tumor tissue RNA-seq was available for each patient, and normal tissue RNA-seq was available for 36 patients.

eBC patients were classified according to IHC subtypes using HR and human epidermal growth factor 2 (HER2): HR^+^HER2^−^, HER2 amplified, and TN (43). Additional stratifications were made using ER^+^ tumor intrinsic subtypes (18) for HR^+^HER2^−^ cases: Lum A, Lum B, basal-like (BL), HER2-enriched (HER2), and for TN intrinsic subtypes (17): BLIA, BLIS, LAR, and MES for TN cases (17).

DFI and OS were evaluated at 5 years. Patients who were alive with no progression for DFI and alive for OS at 5 years were censored.

### HERV detection and selection

HERVs sequences were detected and quantified using Telescope software (44). Only HERVs sequences detected with more than five reads per patient and expressed for at least 5% of patients were considered for further analysis. With this filtering, 14,800 HERVs were detected.

For further analysis, HERVs expressed only in tumor tissue or significantly overexpressed in tumor tissue compared to normal tissue were selected. For this purpose, differential analysis was performed based on HERVs expressed in both normal and tumor tissues and using the DESeq2 R package (45). HERVs were considered overexpressed if the following conditions were met: Benjamini–Hochberg adjusted *p*-value < 0.05 and log2(fold change) > 1.

### Overall HERV tumor load

For each patient, overall HERVs expression was computed by the mean expression of the selected HERVs, hereafter named “overall HERV tumor load”.

### Pathways and transcriptomic signature expression

MCP-counter abundance of 10 tissue-infiltrating immune and stromal cell populations [CD3^+^ T cells, CD8^+^ T cells, cytotoxic lymphocytes, NK (natural killer) cells, B lymphocytes, monocytic lineage, myeloid dendritic cells, neutrophils, endothelial cells, and fibroblasts] was estimated using transcriptomic profiles (46).

Four transcriptomic signatures related to T-cell immune infiltrate were estimated by taking the mean expression of corresponding genes: Cytotoxicity (CYTOX), TH1 orientation (TH1), Cytotoxic Lymphocytes (CTL), and Immune checkpoints and modulators (ICK) (**Supplementary Table 2**).

In previous work, a 40-gene signature specific to lymphocyte, myeloid, stromal, and cancer cells was generated (22). The four cell-type scores were computed by averaging the 10 selected genes representative of the cell type for each sample. TIL score was defined as the ratio of the sum of lymphoid and myeloid scores to the total of lymphoid, myeloid, stromal, and cancer scores.

All these signatures are hereafter named Immune Signatures (IS).

Moreover, expression of Hallmarks of cancer gene sets and three IFN pathways (GOBP RESPONSE TO INTERFERON BETA, REACTOME INTERFERON ALPHA BETA SIGNALING, and GEISS RESPONSE TO DSRNA UP) from MSigDB (https://www.gsea-msigdb.org/gsea/msigdb, accessed on 24 April 2023) was evaluated using single-sample Gene Set Enrichment Analysis (ssGSEA) (47).

### “Breast 19 HERVs” signature

HERV expressions were normalized using the variance stabilizing transformation to eliminate possible technical biases (DESeq2 R package).

To compute the “Breast 19 HERVs” signature, correlations of each selected HERV with the three transcriptomic IFN pathways were estimated. HERVs significantly correlated with all of these pathways in each IHC subtype were selected to estimate a HERVs Breast signature using their average expression.

### Statistical analyses

HERV expressions were compared according to IHC subtypes, ER^+^, and TN tumor intrinsic subtypes using the Wilcoxon test. *p*-values were adjusted using Benjamini–Hochberg FDR correction, and adjusted *p*-values < 0.05 were considered significant. Correlations between HERV expression, immune signatures, and pathways were assessed using Pearson correlation coefficients. Survival analysis was performed using the survival R library. The prognostic value of the different variables was tested in univariate or multivariate Cox models when conditions of the model validity were met. Proportional hazards assumptions were tested based on Schoenfeld residuals. *p*-values < 0.05 were considered statistically significant.

Statistical analyses were performed using R software version 4.1.3 (http://www.R-project.org/) and graphs were drawn using GraphPad Prism version 9.0.2.

## Data Availability

Publicly available datasets were analyzed in this study. This data can be found here: TCGA.

## Supplementary material

Supplementary Figure 1 **(A–D)**. Heatmaps of the expression of HALLMARK signaling pathways and three interferon pathways. Columns represent patients, and rows represent pathways; patients are ordered according to their overall HERV tumor load expression (increasing order, from white to blue) for **(A)** the whole cohort, and according to **(B)** IHC subtypes, **(C)** triple-negative intrinsic subtypes and **(D)** ER^+^ tumor intrinsic subtypes.

Supplementary Figure 2 Venn diagram showing genes significantly correlated with the three interferon pathways that overlapped across each ER^+^ tumor intrinsic subtypes.
