# Supplementary figures and images for: An expression signature of 19 human endogenous retroviruses identifies immunogenic luminal breast cancers likely to respond to immunotherapy
Source: Front Oncol. 2026 Jun 16;16:1728115. doi: 10.3389/fonc.2026.1728115 (PMC13314411; doi:10.3389/fonc.2026.1728115)

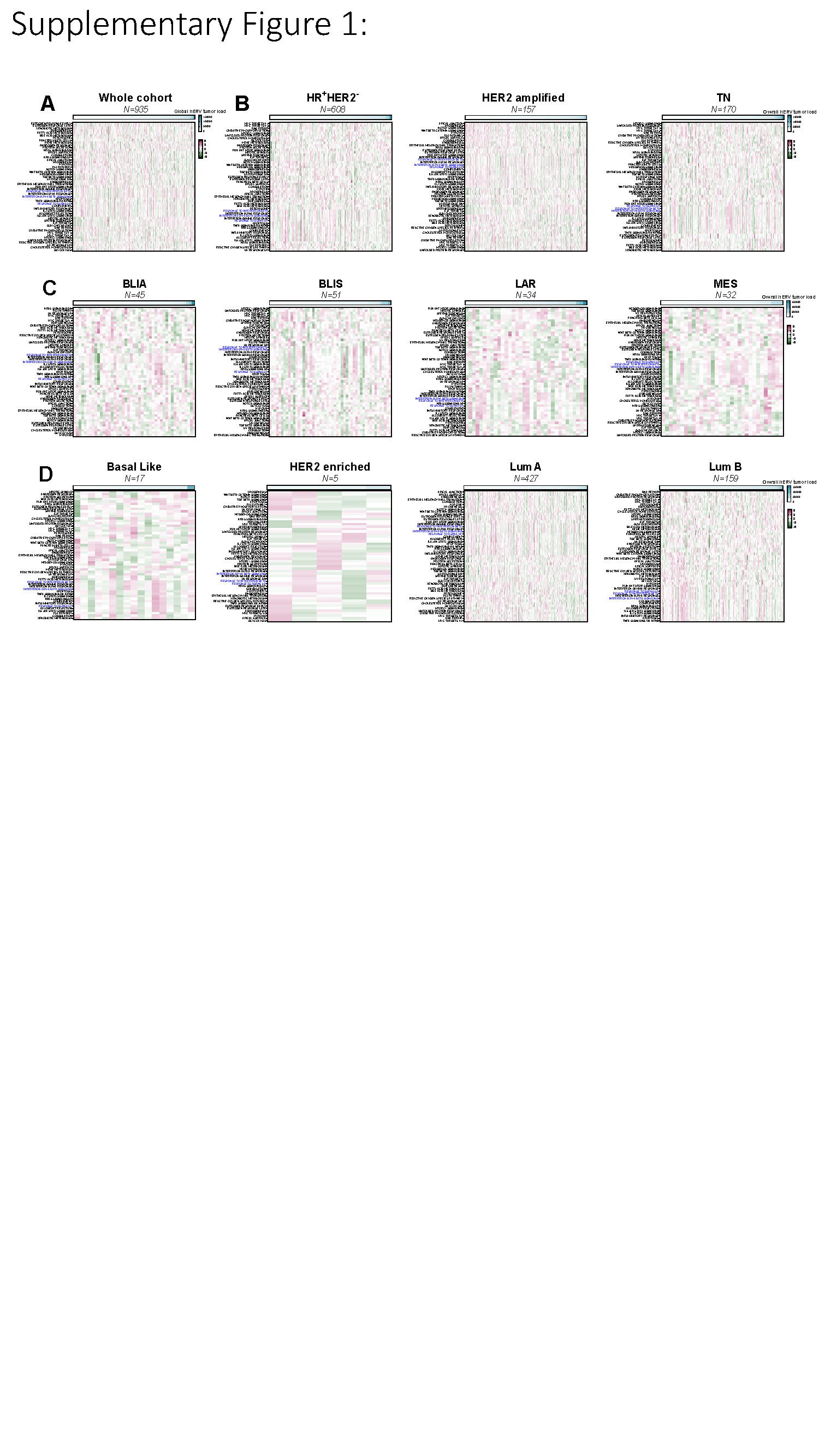

Supplement: Supplementary Figure 1 — (A–D). Heatmaps of the expression of HALLMARK signaling pathways and three interferon pathways. Columns represent patients, and rows represent pathways; patients are ordered according to their overall HERV tumor load expression (increasing order, from white to blue) for (A) the whole cohort, and according to (B) IHC subtypes, (C) triple-negative intrinsic subtypes and (D) ER+ tumor intrinsic subtypes. [file Image1.tiff]

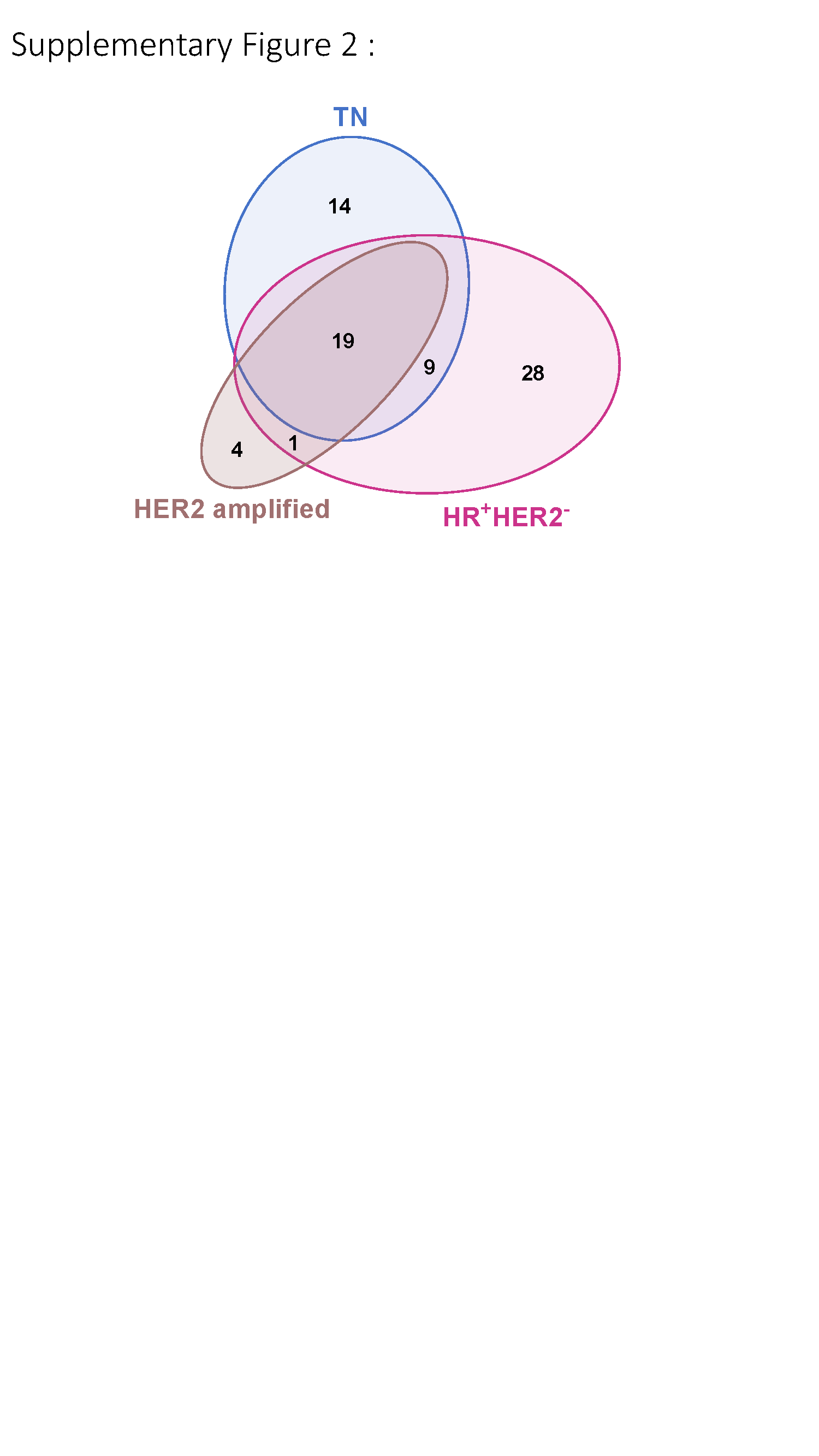

Supplement: Supplementary Figure 2 — Venn diagram showing genes significantly correlated with the three interferon pathways that overlapped across each ER+ tumor intrinsic subtypes. [file Image2.tiff]
