# Supplementary material for: An expression signature of 19 human endogenous retroviruses identifies immunogenic luminal breast cancers likely to respond to immunotherapy
Source: Front Oncol. 2026 Jun 16;16:1728115. doi: 10.3389/fonc.2026.1728115 (PMC13314411; doi:10.3389/fonc.2026.1728115)
Supplement: Supplementary file 3 [file Table1.docx]

**Supplementary Table 1:** Cox models according to the 19 hERVs selected for “Breast hERV19” signature for disease-free interval in whole cohort and in patients with HR^+^HER2^-^ tumors

| **hERV** | **Whole cohort** | **HR^+^HER2^-^** |
| --- | --- | --- |
| HERVW_5p14.3 | 0.8 [0.7, 1]; 0.056 | 0.8 [0.6, 1]; 0.2 |
| ERV316A3_Xp22.11 | 1 [0.9, 1.3]; 0.6 | 1 [0.8, 1.3]; 0.9 |
| ERV316A3_2q36.2 | 0.8 [0.7,1]; **0.045** | 0.7 [0.6, 1]; **0.03** |
| MER4B_19q13.42b | 0.9 [0.7, 1 .1]; 0.3 | 0.7 [0.5, 1]; **0.045** |
| HERV4_5q33.1 | 0.9 (0.7, 1.1]; 0.2 | 1 [0.7, 1.3]; 0.7 |
| HML6_4q21.1 | 0.8 [0.7, 1.1]; **0.01** | 1 [0.8, 1.2]; 0.7 |
| ERVLB4_2q37.1a | 0.8 [0.7, 1]; 0.08 | 0.8 [0.6, 1.1]; 0.2 |
| HERVH_12p13.1b | 1 [0.8, 1.2]; 0.9 | 0.8 [0.6, 1.1]; 0.13 |
| HERVL40_4q32.3b | 0.7 [0.6, 0.9]; **0.01** | 0.8 [0.6, 1.1]; 0.2 |
| MER41_6p22.3 | 0.9 [0.7, 1.1]; 0.2 | 0.9 [0.7, 1.1]; 0.3 |
| HERVEA_5q22.2 | 0.9 [0.8, 1]; 0.06 | 0.8 [0.7, 1]; **0.02** |
| HARLEQUIN_1q32.1 | 0.9 [0.8, 1]; 0.06 | 0.8 [0.6, 0.9]; **0.001** |
| HERVE_1p36.12 | 0.9 [0.8, 1.1]; 0.5 | 0.9 [0.7, 1.1]; 0.2 |
| MER4B_19q13.42a | 0.9 [0.8, 1.1]; 0.2 | 0.8 [0.7, 1]; 0.6 |
| ERV316A3_3q13.31b | 0.9 [0.8, 1]; 0.2 | 0.9 [0.7, 1]; 0.1 |
| HUERSP3_6p21.32 | 0.9 [0.8, 1.1]; 0.3 | 0.9 [0.7, 1.2]; 0.5 |
| HERVH_1q24.2 | 0.9 [0.8, 1]; 0.07 | 0.9 [0.7, 1.1]; 0.2 |
| HML3_8q24.13 | 1 [0.8, 1.1]; 0.6 | 0.7 [0.6, 1.1]; 0.1 |
| ERV316A3_2q22.3c | 1 [0.8, 1.1]; 0.4 | 0.9 [0.7, 1.1]; 0.4 |

Results are described as Hazard Ratio (HR), with 95% confidence interval (CI) and p-value (p); (HR [CI]; p).
