# Supplementary material for: An expression signature of 19 human endogenous retroviruses identifies immunogenic luminal breast cancers likely to respond to immunotherapy
Source: Front Oncol. 2026 Jun 16;16:1728115. doi: 10.3389/fonc.2026.1728115 (PMC13314411; doi:10.3389/fonc.2026.1728115)
Supplement: Supplementary file 4 [file Table2.docx]

**Supplementary Table 2:** Details of genes used in transcriptomic signatures.

| Transcriptomic signatures | Genes |
| --- | --- |
| Cytotoxicity (CYTOX) | GZMA/B/K/H, PRF1 |
| Th1 orientation (TH1) | TBX21, IFNG |
| Cytotoxic lymphocytes (CTL) | CD8A, CD3D/E/G, PTPRC |
| Immune checkpoint and modulators (ICK) | CD40, CD274, ICOS, LAG3, IL2RB, HAVCR2, TNFRSF4, TNFRSF9, TNFRSF18, CD276, CTLA4, PDCD1LG2, VTCN1, PDCD1, BTLA, CD28, C10orf54, CD27, IDO1 |
